# Other better versus self better in baboons: an evolutionary approach of social comparison

**DOI:** 10.1098/rspb.2017.0248

**Published:** 2017-05-24

**Authors:** F. Dumas, J. Fagot, K. Davranche, N. Claidière

**Affiliations:** 1 Nîmes University, CHROME EA7352, Rue du Docteur Georges Salan, 30021 Nîmes Cedex 1, France; 2 Aix Marseille Univ, CNRS, LPC, FED3C, Marseille, France

**Keywords:** social comparison, non-human primates, evolution, social facilitation, baboons

## Abstract

Comparing oneself with others is an important characteristic of human social life, but the link between human and non-human forms of social comparison remains largely unknown. The present study used a computerized task presented in a social context to explore psychological mechanisms supporting social comparison in baboons and compare major findings with those usually observed in humans. We found that the effects of social comparison on subject's performance were guided both by similarity (same versus different sex) and by task complexity. Comparing oneself with a better-off other (upward comparison) increased performance when the other was similar rather than dissimilar, and a reverse effect was obtained when the self was better (downward comparison). Furthermore, when the other was similar, upward comparison led to a better performance than downward comparison. Interestingly, the beneficial effect of upward comparison on baboons' performance was only observed during simple task. Our results support the hypothesis of shared social comparison mechanisms in human and non-human primates.

## 1. Introduction

The present study addresses the crucial but overlooked issue of social comparison [1] (i.e. self-evaluation relative to others) and especially its consequences in non-human primates. The extensive research in humans has demonstrated that comparing oneself with others is ‘an almost inevitable element of social interaction’ [2, p. 150], which occurs spontaneously whenever one is exposed to information about others [3,4]. Either deliberately and actively searched for or imposed by the social context, social comparison influences individuals' emotions, self-evaluations, motivations or behaviours in important ways [5,6]. Research has demonstrated that consequences of social comparison greatly depend on its direction, namely whether one compares with a more or less fortunate other (termed upward and downward comparisons, respectively), and the similarity between oneself and the other on salient characteristics (e.g. category membership [7]; psychological closeness [8]; sex [9]; distinct attribute [10]). Social comparison with a similar other generally results in assimilation, whereas comparison with a dissimilar other leads to a contrast effect [11,12]. Therefore, when the other is similar, upward comparison is likely to lead to positive effects and downward comparison to negative effects (assimilation process), while a reverse pattern is expected in the case of dissimilarity (contrast process).

Because of the adaptive value of adequately sizing up one's competitors to both own survival and group functioning, comparing oneself to others is likely to be phylogenetically ancient and shared by many species [13]. There is some evidence that animals are sensitive to social comparison and can modify their behaviour accordingly. For instance, in the guppy (*Poecilia reticulata*), a species in which a male's reproductive success is influenced by his attractiveness to females, males prefer females surrounded by other males that are less colourful than they are themselves, and the magnitude of this preference is negatively correlated with the male's own level of colour ornamentation [14] (however, cf. [15]). Other interesting findings come from experimental studies on inequity aversion focusing on how animals respond to getting less than a partner [16,17], most often conducted among non-human primates. In the typical paradigm, two individuals from the same social group alternatively exchange some tokens with a human experimenter to receive a food reward. Each can see the other's behaviour and the other's outcomes. In the baseline condition, rewards are the same, but in the inequity condition, one partner receives a more preferred reward than the other. When their conspecific receives a more preferred food (e.g. grape) for equal effort, not only do chimpanzees refuse their low-valued food (e.g. cucumber) but they also refuse to participate altogether [18].

According to Hopper *et al*. [18], this sensitivity to disadvantageous inequity is driven by social comparison (what animals have received in relation to what their test partner has received). These findings seem to indicate that upward comparison results in negative effects (here task disengagement). However, the evidence for inequity aversion among non-human primates are strongly contested (see, for instance, [19,20]; for an overview of successful and failed replications, see [21]). Furthermore, social comparison and inequity aversion could be completely different processes with inequity aversion resulting, for instance, from frustration effects [22,23]. Moreover, social comparison can arise when a discrepancy between oneself and others exists, either to the advantage or disadvantage of the self, and without any inequity of treatment. Thus, inequity aversion is one form of social comparison, and not necessarily the most common nor the most studied form in human social comparison research. Although experiments on inequity aversion suggest that social comparison might exist in non-human animals, they cannot be taken as definitive evidence of it; furthermore, they leave unknown the role of the similarity between the individuals.

An important step in the study of social comparison was made recently with the study of Schmitt *et al*. [24], which provided, to our knowledge, the first and most direct test of social comparison in non-human animals, and demonstrated that social comparisons influence performance in monkeys. Long-tailed macaques (*Macaca fascicularis*) were tested in co-acting paradigm, and an auditory feedback about the alleged performance of the partner was provided via playback to manipulate social comparison. Two factors were used to manipulate similarity—the extremity of the partner (either moderately versus extremely better or worse than the subject) and the relationship quality (mainly based on grooming)—to classify partners as socially close versus distant. Contrary to expectations, dissimilarity (not similarity) with the partner led to assimilation. When tested with a dissimilar (distant) partner, long reaction times (RTs) occurred more frequently when the partner was performing worse rather than better than the subject. These unexpected findings led Schmitt *et al*. to conclude that monkeys do not share the specific social comparison processes resulting in assimilation and contrast effects in humans, and that the elaborate social comparison processes found in humans may be ‘a derived feature of our own species’ [24, p. 427]. However, such a conclusion seems premature, and the present study aims to extend this first study in three important directions.

First, sex is one of the most important self-defining attributes common to both human and non-human primates, and is therefore particularly relevant for social comparison processes [25–28]. Contrary to Schmitt *et al*. [24], who neither manipulated nor controlled for sex category, we used this major feature as a key variable defining similarity.

Second, Schmitt *et al*. [24] did not consider task complexity in their study. A great deal of research in social facilitation has demonstrated that the presence of conspecifics (present as co-actors or passive audience) produces an increase in general arousal, which in turn improves performance on easy or well-learned tasks and impairs performance on difficult or poorly learned tasks [29] (see [30] for a review). Our purpose was also to examine how the level of performance of a partner (a better-off or a worse-off other) influenced the subjects' performance in relation to both the similarity between the subject and its partner and the complexity of the task.

Finally, the last important goal of the present research was to allow a more direct comparison with experimental research on humans. Tesser *et al*.'s [8] landmark experiment is, to our knowledge, the only one to have tested the effects of social comparison in humans (upward versus downward comparison provided through computerized feedback) as a function of both similarity (friend versus stranger) and task complexity (entering a single randomly selected digit five times versus five different digits on a computer). Tesser *et al*. predicted and found that upward comparison with a similar other rather than a dissimilar other led to a higher performance (i.e. faster RTs) on a simple task and a lower performance (i.e. longer RTs) on a complex task. Additionally, when the partner was similar, upward comparison relative to downward comparison led to a higher performance (i.e. faster RTs) on a simple task and a lower performance (i.e. longer RTs) on a complex task. To maximize the relevance of friendship, Tesser used only same-sex pairs by excluding male participants. Thus, we used Tesser *et al*.'s [8] results to guide our analysis, and we predicted that the same pattern of results should emerge in our study if the baboons shared similar social comparison processes with humans.

## 2. Material and methods

This study used a large existing dataset describing contextual cueing effects in baboons. In their study, Goujon & Fagot [31] studied how 21 baboons differentially learn to find a target among a set of distractors that were either predictive of the target location or non-predictive. Here, we used the response of the baboons when they were in the presence of exactly one partner to study the effect of social comparison on performance (a total of 147 387 trials). This very large sample size, both in terms of the number of primates studied and in terms of the number of trials performed, allows for the study of complex interactions between predictor variables. In the following, we present only the most relevant aspects of the contextual cueing experiment; more details can be found in the original study of Goujon & Fagot [31].

### (a) Subjects and living conditions

Twenty-one Guinea baboons (*Papio papio*) belonging to a large social group of the CNRS Primate Center in Rousset-sur-Arc (France) participated in this study. They were 5 males (mean age 5.4 years, s.d. = 3.0 years) and 16 females (mean age 8.5 years, s.d. = 5.3) with ages ranging from 2 to 17. The baboons were all marked by two biocompatible 1.2 by 0.2 cm radio frequency identification (RFID) microchips injected into each forearm.

### (b) Self-testing apparatus

**Figure 1.**
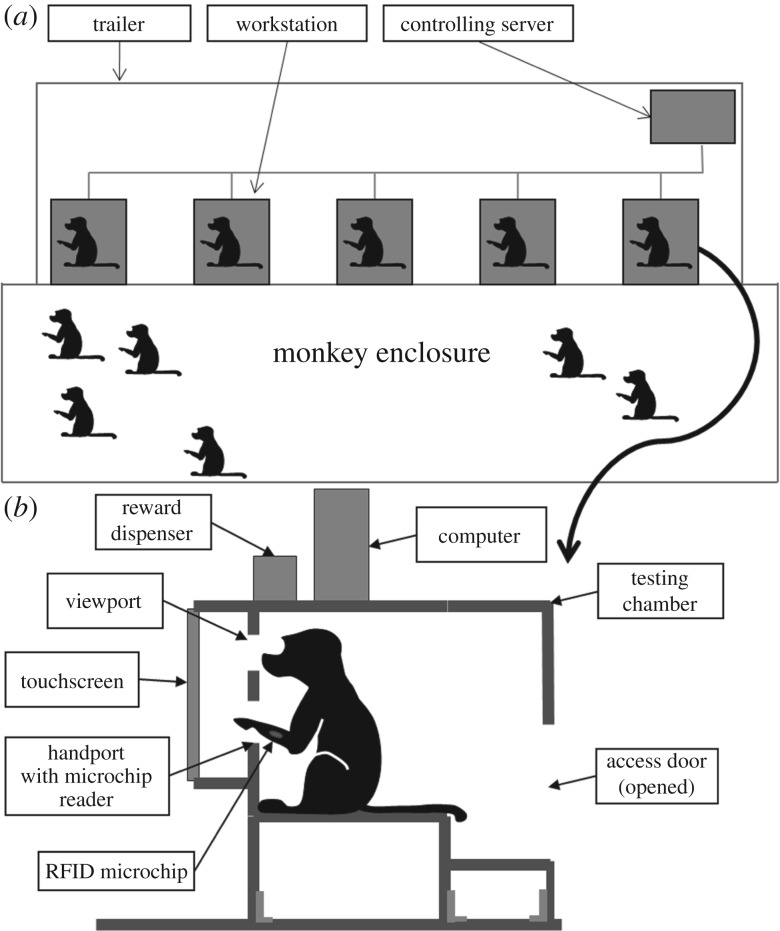
Principle of the self-testing apparatus. (*a*) Bird's-eye view of the enclosure and the trailer containing the workstations. (*b*) Schematic of a baboon working at a workstation. (Online version in colour.)

The study was conducted in a unique testing facility developed by Fagot & Bonté [32]. The key feature of this facility is that baboons have free access from their 20 × 30 m enclosure to 10 computerized testing booths that are installed in trailers next to their enclosure (see figure 1). Each workstation comprises a test chamber, with transparent side walls, that can be opened at the rear. The front of the test chamber is fitted with a view port (7 × 7 cm) and two hand ports (8 × 5 cm). Looking through the view port allows visual access to a 19-inch LCD touch monitor installed at eye level 25 cm from the view port. Two antennae, fixed around each arm port, read the RFID identity of an animal when one of its forearms is introduced through one of the two arm ports. Identification signals from the microchip are used by the computer to trigger the presentation of the stimulus and to assign behavioural measures to each participant. The equipment is controlled by a test program written with Eprime (Psychology Software Tools, Pittsburgh, PA). The test program allows an independent test regimen for each baboon, irrespective of the test chamber it is using [33]. Grains of dry wheat are used as a reward (for more details, see [32,33]). During this experiment, the monkeys could see their partners working in adjacent workstations but were unable to see their motor responses on the screen; observational learning was thus impossible.

### (c) Experimental procedure

The contextual cueing task consisted in finding a target on a touchscreen containing several distractors (stimuli and data have been posted in an open access repository: https://osf.io/8ct3r; doi:10.17605/OSF.IO/8CT3R). Testing occurred during one full month. During that period, the baboons continually received blocks of 12 test trials. Two levels of difficulty were used. Each block included six predictive trials and six shuffled trials. Six configurations never used in training were assigned to the predictive condition and six to the shuffled condition. The configurations were counterbalanced among subjects. Each predictive configuration was associated with a constant target location. Predictive trials were therefore easy trials because the visual search could be guided by the predictive background. In the more difficult shuffled configurations the target was shown with six different backgrounds, but the location of the target was independent of the background. Altogether, the baboons received an average of 7369 trials in the task (range 300–11 762, s.d. = 2445).

Since the animals were not captured during the experiment, the social context in which they performed the computerized task varied spontaneously. Thus, on some trials, the baboons used the computers with no conspecific nearby, whereas on other trials one or two animals were present in adjacent workstations. This innovative procedure allowed us to remove any bias associated with the stress of social deprivation [34], as inferred from the significant decrease in salivary cortisol as well as the frequency of stereotypies, which is generally not the case in social facilitation studies [30]. Our experimental procedure based on a voluntary participation of the subjects reduces stress, as inferred from the significant decrease in salivary cortisol as well as the frequency of stereotypies [35]. As indicated previously, for the purpose of the current paper, only the subject's trials performed with exactly one adjacent conspecific were used.

### (d) Data analysis

We analysed the results using generalized linear mixed models (GLMM) and followed the procedure recommended by Zuur *et al*. [36]. Our dependent variable was RTs (in ms) of correct trials (the success rate in this experiment was uniformly high, we therefore chose to ignore unsuccessful trials) of baboons with exactly one neighbour. Based on previous work, we knew that younger individuals tended to respond faster than older ones [37] and also that in this particular study there was a clear decrease in RTs with the progress of the study (i.e. a learning effect; see [37]). Therefore, we chose to include the age of the baboons and the number of days since the study started as random effects.

Our analysis focused on reproducing the effects described in Tesser *et al*.'s [8] experiment, which concern the modulations in response time depending on task complexity, the direction of social comparison and the similarity with the comparison target. Accordingly, we included three explanatory variables in our analyses and their interaction. The first variable represented the task complexity (simple versus complex). We used the difficulty of the task either predictive or shuffled that we knew had a strong effect on RTs [31]. The second variable represented the similarity between the subject and its partner (same sex versus different sex). Finally, the last variable aimed at measuring the direction of comparison (upward versus downward). We had two options regarding this last variable, one consisting in manipulating the performance of the baboons by, for instance, randomly attributing an easy or a difficult task to different individuals and by analysing the effect of the success or failure of one individual on their neighbour. However, if individuals have knowledge of each other's capacities and performances, this knowledge can potentially interfere with arbitrary manipulations of performance to produce results that are difficult to interpret (for instance if a very good individual is repeatedly failing). Furthermore, in our set-up the baboons cannot directly observe the task given to the other baboons and their responses on the touch screen. The only feedback the baboons can get comes from the observation of the other individual being rewarded (they can see other baboons picking up rewards and eating). Accordingly, we chose to use the difference in average number of rewards obtained by the focal baboon and its partner in the month preceding the experiment, as a measure of perceived difference in performance. This measure has the advantage of providing a realistic measure of the difference that the baboons might perceive between its own and the other's performance. However, it also represents one limit of our study because it cannot inform us on the direct influence of the success or failure of a neighbour (this choice is discussed further in the Discussion section).

We used an AICc-based model selection approach in which we fitted 12 possible models produced from the three explanatory variables. We present the results of the best fitting model and conducted only a limited number of planned comparisons in relation to the hypothesis formulated based on the human literature; we therefore report exact *p*-values.

## 3. Results

Table 1 summarizes the AICc scores of a total of 12 different possible models and shows that the only model supported by the data contains the predicted three-way interaction among the task complexity (complexity), direction of social comparison (comparison) and similarity factors (similarity).

**Table 1.** Model comparison table. Among all possible models, the best model supported by the evidence (with an AICc weight greater than 0) includes a three-way interaction between the complexity of the task, the direction of social comparison and similarity. K stands for the number of parameters, LL for log-likelihood, AICc for the corrected Akaike information criterion, ΔAICc for the difference in AICc between the current model and the best-fitting model, and AICcWT for the corrected weight of evidence that supports the model. The models are ordered according to their AICc.

model	*K*	LL	AICc	ΔAICc	AICcWt
complexity × comparison × similarity	13	−986 675.3	1 973 377	0	1
complexity × comparison	9	−986 689.7	1 973 397	20.65	0
complexity × comparison + similarity	10	−986 689	1 973 398	21.35	0
complexity	7	−986 696.7	1 973 407	30.74	0
complexity + comparison	8	−986 695.7	1 973 407	30.76	0
complexity + similarity	8	−986 695.8	1 973 408	30.87	0
complexity + comparison + similarity	9	−986 695.1	1 973 408	31.43	0
complexity × similarity	9	−986 695.2	1 973 408	31.67	0
complexity × similarity + comparison	10	−986 694.5	1 973 409	32.23	0
similarity	7	−988 174.2	1 976 362	2985	0
comparison	7	−988 173.8	1 976 362	2985	0
intercept	6	−988 175.1	1 976 362	2985	0

To analyse the three-way interaction of the best-fitting model, we fix the continuous variable comparison at two extreme values, a positive difference equal to the mean plus two standard deviations between individuals corresponding to the downward comparison condition and a negative difference equal to the mean minus two standard deviations, corresponding to the upward comparison condition. This is justified by the fact that our predictions apply when individuals can perceive differences in performance, which is possible only when a certain difference is achieved. Furthermore, the values used to determine the effects are realized in the population (the difference in performance varied from a minimum of −0.17 to a maximum of 0.18 for an average of 0.00 and a s.d. of 0.057).

**Figure 2.**
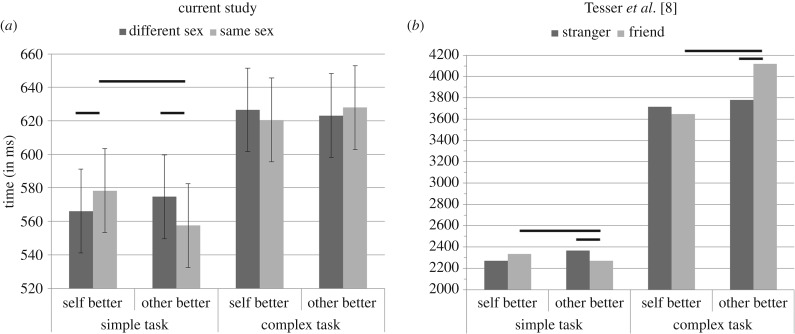
(*a*) Estimated differences in reaction times from the averaged model for the three explanatory variables, task complexity (simple versus complex), comparison (downward/self better versus upward/other better) and similarity (same sex versus different sex). Error bars represent standard errors. Horizontal bars indicate a significant difference between the two conditions. (*b*) For comparison purposes, this graph illustrates the main results of Tesser *et al*.'s study on social comparison effects in humans [8].

Figure 2 shows that the direction of change in every condition corresponds to the predictions formulated on the basis of results in humans. In easier task (predictive trials), when the partner is better (upward comparison), there is a performance improvement with similarity (RTs decrease by an estimated 17.2 ms, *t* = −4.834, *p* < 0.001, 95% CI = [10.1, 24.3]), opposite results are observed when the self is better (downward comparison: RTs increase by 12.2 ms, *t* = 3.52, *p* < 0.001, 95% CI = [5.3, 19.1]). When the task becomes more difficult (non-predictive trials), no significant changes are observed in upward comparison (*t* = −1.34, *p* = 0.18, 95% CI = [−2.3, 11.9]), but there is a marginally significant performance improvement in downward comparison (RTs decrease by 6.0 ms, *t* = 1.72, *p* = 0.085, 95% CI = [−0.86, 12.9]).

Furthermore, as predicted, in the same-sex condition better performance is reported on the simple task when the partner is better, compared with when he/she is poorer (RTs decrease by an estimated 20.8 ms, *t* = 4.91, *p* < 0.001, 95% CI = [12.5, 29.1]). On the complex task, the pattern found is the opposite of the one found in the simple task, but it does not reach significance. In the same-sex condition, there is only a marginally significant decrease in performance when the partner is better compared to when he/she is poorer (RTs increase by an estimated 7.4 ms, *t* = −1.73, *p* = 0.08, 95% CI = [−0.93, 15.7]).

## 4. Discussion

The present findings demonstrate for the first time that the consequences of social comparison can be similar among human and non-human primates. Figure 2, which juxtaposes our results with those of Tesser *et al*. [8], shows how striking the parallel is. In line with Tesser *et al*., we observed a three-way interaction between similarity (same sex versus different sex), comparison direction (upward versus downward) and task complexity (simple versus complex). When the partner was similar and the task was simple, upward comparison led to positive effects and downward comparison to negative effects (assimilation process). A reverse pattern was observed in case of dissimilarity (contrast process). Although only marginally significant (probably due to a floor effect), the reverse pattern was obtained on the complex task. These findings are important. They provide evidence that social comparison is shared with other non-human primates with similar consequences on performance. This also suggests that social comparison in non-human primates and humans relies on psychologically similar processes that have evolved to serve similar functions.

Our findings contrast with those of Schmitt *et al*. [24], who showed an effect of social comparison on performance among non-human primates but obtained different results on assimilation and contrast effects as a function of similarity versus dissimilarity of the comparison target. According to the authors, the lack of effect in their study could be due to the fact that the direction of the comparison among monkeys was signalled by the distribution of a reward, and not simply by the difference in performance as in humans. However, our study also used a reward-based reinforcement procedure and led to the expected effects. At least three reasons could explain the difference between our results and those of Schmitt *et al*. [24]. First, sex is probably a more salient, more powerful and more stable factor of similarity than the composite sociality index used by Schmitt *et al*. Previous research in humans highlighted the importance of the sex membership in social comparison behaviours [25,28]. Our findings confirm for the very first time that sex is also a decisive attribute for social comparison in non-human primates. Second, the present findings are in line with more than 50 years of research on a variety of animal species (from cockroaches to non-human and human primates) demonstrating the importance of task complexity for social facilitation. The fact that assimilation and contrast effects depended not only on similarity (between the subject and the comparison other), but also on task complexity, represents a significant contribution of the present study to research on social comparison in animals.

Finally, a unique feature of our facility is that the baboons tested here have a long-standing experience of the experimental area and are used to working in the presence of one or several co-actors. Therefore, we could use the real discrepancy in average number of rewards obtained by the subject and its partner in the month preceding the experiment as a proxy to evaluate the direction of social comparison. This measure reflects real cognitive differences and does not provoke conflicting information between the task and the subject knowledge of the partner's performance. However, this reliance on realized performances also represents a weakness of our study since we did not directly manipulate the perceived differences in performances on a trial-to-trial basis. However, other studies of social comparison using coaction settings with humans have also relied on an overall difference in performance [38,39]. For example, in experiment 2 of Huguet *et al*. [38], participants were forced to compare themselves with a confederate during the experimental session preceding the focal task measuring their performance. The authors found a beneficial effect of upward comparison on the focal task. In other words, the social comparison was not an effect of one trial over another, but was induced by an overall difference in performance during a previous session.

In our opinion, future experiments that seek to test the direct effect of the success/failure of another individual on performance (with or without the experimental manipulation of success/failure) need to take into account the fact that the individuals are familiar with each other and would benefit greatly from a more direct exposure to the performance of the other individual (with the individual getting a direct visual experience of the task the other individual is performing). In any case, a comprehensive picture of social comparison in non-human primates necessarily involves these complementary approaches.

There is no doubt that humans socially compare in more complex ways than other animals, including baboons. The guinea baboons tested in this study are a highly tolerant and cooperative species [40]. Therefore, the present findings provide further evidence in favour of the view that social comparison represents a specific adaptation to cooperative group living [41,42]. An interesting extension of the current research would be to investigate whether social comparison in non-human primates is driven by arousal and/or by more complex mechanisms involving attention. For instance, recent findings in baboons showed that the presence of conspecifics consumed cognitive control resources that are required for successful performance [43]. In more general terms, our results demonstrate both the complexity and the flexibility of social comparison processes at work in animals and contribute to a growing literature demonstrating the importance of considering the social context when assessing the performance of animals in behavioural and cognitive studies.
